# Optimal Localization of the Foramen Ovale for Transseptal Puncture Using the Vertebral Body Units

**DOI:** 10.3390/medicina61050896

**Published:** 2025-05-15

**Authors:** Dong Hoon Kang, Sung Eun Park, Jong Woo Kim, Seong Ho Moon, Ho Jeong Cha, Jong Hwa Ahn, Joung Hun Byun

**Affiliations:** 1 Department of Thoracic and Cardiovascular Surgery, Gyeongsang National University College of Medicine and Gyeongsang National University Changwon Hospital, Changwon 51472, Republic of Korea; drk82@hanmail.net (D.H.K.); hoya_m@naver.com (S.H.M.); nombrehj@gmail.com (H.J.C.); 2 Department of Radiology, Gyeongsang National University College of Medicine, Changwon 52828, Republic of Korea; uneyes@hanmail.net; 3 Department of Cardiology, Gyeongsang National University College of Medicine and Gyeongsang National University Changwon Hospital, Changwon 51472, Republic of Korea; jonghwaahn@naver.com

**Keywords:** transseptal puncture, chest computed tomography scout view, chest X-ray, foramen ovale position, vertebral body unit

## Abstract

*Background and Objectives*: Although transesophageal or intracardiac echocardiography and radiofrequency needles are employed to guide transseptal puncture, their routine utilization is associated with substantial expense. No reports have analyzed the use of the foramen ovale position to effectively guide transseptal punctures on chest X-rays or computed tomography scout views, which are more cost-effective approaches to safely and effectively guide the procedure. We aimed to find the foramen ovale position on chest computed tomography scout views to effectively guide percutaneous transseptal punctures. *Materials and Methods*: The study population included 31 patients treated with extracorporeal membrane oxygenation (ECMO) for cardiogenic shock, 32 patients diagnosed with atrial fibrillation (AF) who underwent MDCT, and 197 patients who underwent MDCT for non-cardiac conditions. Vertebral body units, defined as the distance between two adjacent vertebral bodies (the sixth and seventh thoracic spines) inclusive of the intervertebral disk space, were used to express the distance from the carina to the foramen ovale on computed tomography scout views. *Results*: The mean vertebral body units, distance from the carina to the foramen ovale (carina–foramen ovale), and distance from the carina to the foramen ovale on chest computed tomography scout views (carina–foramen ovale vertebral body units^−1^) were 2.3 ± 0.2 cm, 6.9 ± 0.9 cm, and 3.0 ± 0.3, respectively. Multivariate analysis showed significant correlations between the carina–foramen ovale vertebral body units^−1^ and sex (β = 0.080; *p* = 0.028), body mass index (β = −0.020; *p* < 0.001), age (β = 0; *p* = 0.013), and the application of extracorporeal membrane oxygenation or the presence of atrial fibrillation (β = 0.130; *p* = 0.004). *Conclusions*: Although a three-dimensional approach was not employed, the foramen ovale position may serve as a radiologic guide in various clinical settings where transseptal punctures are required. This technique may be an effective aid in transseptal puncture procedures.

## 1. Introduction

Ross et al. introduced transseptal puncture (TP) into clinical practice in the late 1950s [1] as a diagnostic tool to ensure the proper selection of patients for cardiac valve surgery, and it is an important technique employed by electrophysiologists. Generally, fluoroscopic landmarks are used for TP. More recently, transesophageal (TEE) or intracardiac echocardiography (ICE) have been used to guide TP, allowing for a more precise localization of the TP site [2,3]. Percutaneous TP is often performed for left heart decompression when performing veno-arterial extracorporeal membrane oxygenation (ECMO) for patients with heart failure [4,5]. Despite its utility, TP has a potential for serious complications, such as aortic puncture, cardiac perforation, and systemic emboli [6]. Reports suggest that employing a patent foramen ovale (PFO) during balloon mitral valvuloplasty may lead to reduced procedure times and a lower incidence of significant complications such as hemopericardium and cardiac tamponade, as well as atrial septal defects [7]. However, a persistent limitation lies in the requirement for the precise anatomical localization of the PFO.

It is believed that approximating the location of the foramen ovale (FO) on radiologic views, with the assistance of TEE or ICE, may significantly aid in performing safe and effective TP.

Specifically, our aim is to facilitate transseptal puncture (TP) for most clinicians by utilizing readily available two-dimensional radiologic views in the clinical setting. To the best of our knowledge, no studies have analyzed the optimal FO position for effectively performing TP using chest X-ray or chest computed tomography (CT) scout views; this technique, however, only provides a two-dimensional perspective. Herein, we sought to find the location of the FO using vertebral body units (VBUs) for safe and effective TP. This study demonstrated the use of the VBU, which was defined as the distance between two adjacent vertebral bodies, including the intervertebral disk space, to locate the FO on a chest CT scout view, using the carina as a landmark, for effective and safe TP.

## 2. Materials and Methods

### 2.1. Study Design

This retrospective study was approved by the institutional ethics committee of our institution (approval number: GNUCH 2022-04-011). Given that this was a retrospective study, the informed consent requirement was waived.

### 2.2. Participants

The study population comprised 260 patients who underwent multidetector-row computed tomography (MDCT) at our institution between September 2020 and June 2021. This cohort included 31 patients treated with ECMO for cardiogenic shock, 32 patients diagnosed with atrial fibrillation (AF) who underwent MDCT, and 197 patients who underwent MDCT for non-cardiac conditions. The exclusion criteria included destructive lung disease, a history of heart or lung surgery, and spine disease or a history of spine surgery.

### 2.3. Image Acquisition and Measurement

**Figure 1:**
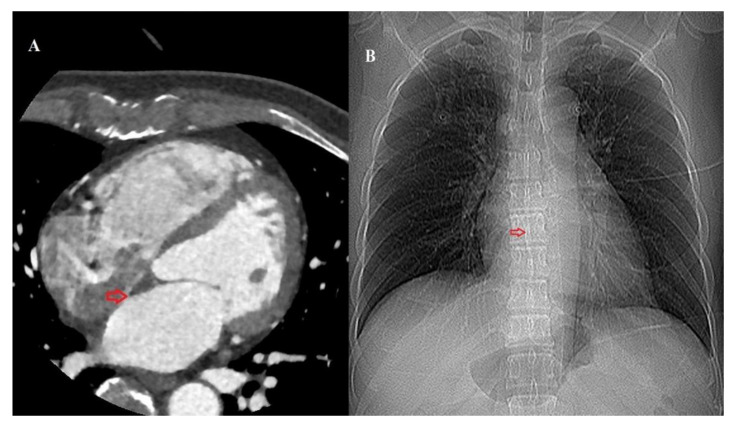
(**A**) Axial multidetector-row computed tomography (CT) image of the heart identifying the fossa ovalis (red arrow). (**B**) The location of the fossa ovalis is marked on the corresponding CT scout image.

All MDCT scans were performed using a third-generation dual-source CT (Siemens Somatom FORCE, Siemens Healthineers, Forchheim, Germany). Iodinated contrast material (200 mL; Iomeron 400; Bracco imaging S.p.A., Milan, Italy) was injected using a power injector at a flow rate of 5 mL/s, followed by a 50 mL saline chaser. The MDCT scans were viewed using commercially available post-processing software linked to the institutional PACS system (Centricity PACS, GE Medical Systems, Milwaukee, WI, USA) for analysis and measurements (Aquaris iNtuition, version 4.4.11, TeraRecon, Foster City, CA, USA). Interdependent multiplanar reconstructions in the primary axial plane, as well as reconstructed four-chamber views, were used to review the data sets. The fossa ovalis was visually identified on MDCT scans as the area on the atrial septum exhibiting the closest contact between the right and left atrial blood pools. This identification was performed by a radiologist with 10 years of experience. On multiplanar reconstructed images, the center of the FO was then marked on the corresponding CT scout image (Figure 1).

**Figure 2:**
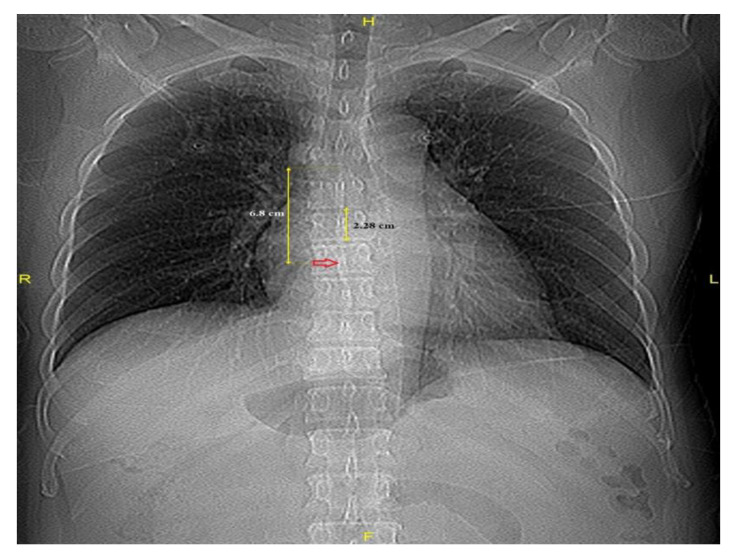
Distances from the carina to the fossa ovalis (red arrow) and a vertebral body unit.

A VBU was defined as the distance between two adjacent vertebral bodies (sixth and seventh thoracic spines), including the intervertebral disk space, and was measured from the inferior endplate of the vertebra. The identification of the carina’s location was achieved through a CT scout view demonstrating the origins of the left and right main bronchi. Using electronic calipers, the distance from the carina to the FO in VBUs on the CT scout view (Carina–FO VBU^−1^) was measured (Figure 2).

### 2.4. Statistical Analysis

Continuous variables are expressed as means ± standard deviations, and categorical variables are presented as absolute numbers and proportions (%). Overall comparisons between groups were performed using Student's *t*-test for continuous variables and the Chi-square test or Fisher’s exact test when the Cochran rule was not met for categorical variables. Relationships between the Carina–FO VBU^−1^ and variables were assessed using univariate and multivariate linear regression analyses. Variables that were statistically significant in univariate regression models (*p*-values < 0.1) were included in a multivariate regression model, and a backward elimination method was used to determine whether they remained significant after adjusting for potential confounders. The odds ratio and 95% confidence intervals were calculated. A two-tailed *p*-value < 0.05 was considered significant.

## 3. Results

### 3.1. Participants

The baseline characteristics of the patients are shown in Table 1.

### 3.2. Outcome Data

We enrolled 260 patients in this study; 150 were male (57.7%), and 110 were female (42.3%). The mean age was 63.8 ± 13.5 years, the mean body surface area was 1.7 ± 0.2 m^2^, and the mean body mass index (BMI) was 24.8 ± 3.9 kg/m^2^ (Table 1). The mean VBU, distance from the carina to the FO (Carina–FO), and Carina–FO VBU^−1^ were 2.3 ± 0.2 cm, 6.9 ± 0.9 cm, and 3.0 ± 0.3, respectively.

Carina-to-FO and VBU measurements according to the application of ECMO and the presence of AF are presented in Table 2. The correlation between Carina–FO VBU^−1^ and the application of ECMO or the presence of AF was strong (*p* = 0.001). Patients with AF had a greater Carina–FO than patients with no cardiac problems or those undergoing ECMO (*p* < 0.001).

Table 3 shows the factors associated with Carina–FO VBU^−1^. Multivariate analysis showed significant correlations between Carina–FO VBU^−1^ and sex (β = 0.080; *p* = 0.028), BMI (β = −0.020; *p* < 0.001), age (β = 0; *p* = 0.013), and the application ECMO or the presence of AF (β = 0.130; *p* = 0.004). The higher the BMI, the smaller the Carina–FO VBU^−1^ value.

## 4. Discussion

Multiple interventional procedures utilizing TP have been developed, including left heart electrophysiology ablations, percutaneous mitral valve repair, patent foramen ovale closure, and left ventricular assist device positioning [8]. TP is generally safe when performed by experienced physicians. However, TP can be technically difficult and cause severe complications, including cardiac tamponade and aortic root puncture [9]. To mitigate these potential risks, we conducted this study with the aim of providing practical guidance for performing TP in clinical settings. Our findings indicate that the location of the FO is approximately three VBUs inferior to the carina.

Verma et al. utilized ICE in addition to fluoroscopic views in all cases for effective and safe TP, visualized the FO on pre-ablation CT, and localized the transseptal needle accurately within the margins of the FO utilizing EnSite Fusion™ and Verismo™ software. With this method, the exact location of the FO can be determined [10]. At our center, we routinely perform TP utilizing both fluoroscopy and TEE.

Utilizing fluoroscopy in the anteroposterior (AP) view is crucial for two key objectives during this procedure: the meticulous monitoring of the needle tip’s position and axial orientation as it enters the FO, and ensuring procedural safety by preventing excessive retraction towards the inferior vena cava [8]. This view is almost identical to the chest CT scout view or the supine chest AP view. Thus, we aimed to find the location of the FO on chest CT and demonstrate the relationship between the location of the FO and vertebrae on the chest CT scout view. There was almost no difference in the positional relationship of chest structures observed on the chest CT scout and AP fluoroscopic views.

In clinical practice, the main role of ICE or TEE is to find the exact location of the puncture site of the procedures, while the role of fluoroscopic views may be to track and place the working catheters. If we already know the approximate position of the FO on the AP fluoroscopic view, the range of movement of the catheters for TP can be narrowed, and the accuracy of the TP can be further improved. In addition, not all TP procedures are performed on the FO on AP fluoroscopic views, but if we know the FO location on the AP fluoroscopic view, it may be easier to find another puncture site on another view. Furthermore, it should be noted that it may be somewhat unreasonable to perform TP with only the aid of the radiologic location of the FO that we have suggested.

We used the VBU as a measurement tool to express the relative position of the FO with respect to the carina; this could offer a more robust assessment, less influenced by variations in body size, rather than representing the absolute position of the FO. Using the vertebrae as a measure to delineate the position of the FO relative to the vertebra has advantages. The vertebra is minimally affected by geometric magnification and is adaptive to somatic growth [11,12]. In addition, the carina has been used as a more reliable landmark because the relative position of the FO should be found on the chest CT scout view, and TP is performed on the AP fluoroscopic view.

In the present study, the distance from the carina to the FO was 6.9 ± 0.9 cm or 3.0 ± 0.3 VBUs below the carina on the chest CT scout view in all patients. The distance from the carina to the FO was 7.5 ± 0.7 cm or 3.2 ± 0.4 VBUs below the carina on the chest CT scout view, particularly in patients with AF. Patients with AF generally have a larger right atrium, meaning that the FO is farther from the carina. We advise that TP should be performed at a lower position in patients with heart problems than in those without heart problems.

In multivariate analysis, factors affecting Carina–FO VBU^−1^ were male sex (β = 0.080; *p* = 0.028), BMI (β = −0.020; *p* < 0.001), age (β = 0; *p* = 0.013), and the application of ECMO or the presence of AF (β = 0.130; *p* = 0.004). A significant correlation was found between the BMI and Carina–FO VBU^−1^. However, the mechanism by which the BMI affects the position of the FO remains unknown.

Typically, the age-related decline in arterial compliance leads to systolic hypertension and left ventricular hypertrophy [13]. Similarly, as the lung elasticity decreases, the stiffness of the chest wall increases and respiratory muscle strength decreases [14]. However, the cause of the increased distance from the carina to the FO with age remains to be investigated. Nevertheless, our study demonstrates a more normative value for TP using the carina and vertebrae as landmarks.

Our study had some limitations. The sample size of this single-center, retrospective study was small. In particular, the number of patients with AF was small. Moreover, our study population was restricted to Asians. As we were limited by the data available in our retrospective study, a large-scale study should be conducted to obtain evidence from other populations and overcome the limitations of the present study. It is our hope that the results of our study, along with our approach to locating the FO, will inform future investigations into other radiologic views, a direction we also intend to explore.

## 5. Conclusions

Although there were some limitations and our technique provides a two-dimensional radiologic view, we anticipate that it could offer helpful guidance for a safer and more effective TP by indicating the location of the FO in a clinically practical manner. In the future, we think that it is necessary to conduct research to find the three-dimensional position of the FO for more accurate anatomic information to guide cardiac procedures.

## Figures and Tables

**Table 1:** Clinical characteristics of patients (N = 260).

Characteristics	N
No cardiac problems (%)	197 (75.8)
- Application of ECMO (%)	31 (11.9)
- AF (%)	32 (12.3)
Male (%)	150 (57.7)
Age (years)	63.8 ± 13.5
Weight (kg)	66.2 ± 13.3
Height (cm)	162.9 ± 9.4
BSA (m2)	1.7 ± 0.2
BMI (kg/m2)	24.8 ± 3.9
DM (%)	63 (24.2)
Hypertension (%)	105 (40.5)

Data expressed as mean ± SD. AF, atrial fibrillation; BMI, body mass index; BSA, body surface area; DM, diabetes mellitus; ECMO, extracorporeal membrane oxygenation.

**Table 2:** Carina-to-FO and VBU measurements according to the application of ECMO and the presence of AF.

	No Cardiac Problems (N = 197)	Application of ECMO (N = 31)	AF (N = 32)	*p*-Value
VBU (cm)	2.3 ± 0.2	2.4 ± 0.2	2.3 ± 0.2	0.002
Carina–FO (cm)	6.8 ± 0.8	7.3 ± 1.2	7.5 ± 0.7	<0.001
Carina–FO VBU−1	3.0 ± 0.3	3.1 ± 0.4	3.2 ± 0.4	0.001

Data expressed as mean ± SD. AF, atrial fibrillation; Carina–FO, distance from the carina to the foramen ovale; Carina–FO VBU^−1^, distance from the carina to the foramen ovale in VBU on the chest CT scout view; CT, computed tomography; ECMO, extracorporeal membrane oxygenation; VBU, vertebral body unit.

**Table 3:** Factors associated with Carina–FO VBU^−1^.

Parameters	Univariate Model		Multivariate Model	
	β (95% CI)	*p*-Value	β (95% CI)	*p*-Value
Male	0.090 (0.010, 0.160)	0.027	0.080 (0.010, 0.160)	0.028
BMI	−0.020 (−0.030, −0.010)	<0.001	−0.020 (−0.030, −0.010)	<0.001
DM	−0.030 (−0.120, 0.060)	0.463		
Hypertension	−0.020 (−0.100, 0.060)	0.647		
Age	0.010 (0, 0.010)	<0.001	0 (0, 0.010)	0.013
Application of ECMO or AF	0.120 (0.030, 0.210)	0.007	0.130 (0.040, 0.210)	0.004

AF, atrial fibrillation; BMI, body mass index; Carina–FO VBU^−1^, distance from the carina to the foramen ovale in VBU by chest CT scout view; CI, confidence interval; CT, computed tomography; DM, diabetes mellitus; ECMO, extracorporeal membrane oxygenation; VBU, vertebral body unit.

## Data Availability

The data presented in this study are available on request from the corresponding author.

## Abbreviations

The following abbreviations are used in this manuscript:

TP	transseptal puncture
TEE	transesophageal
ICE	intracardiac echocardiography
ECMO	extracorporeal membrane oxygenation
FO	foramen ovale
CT	computed tomography
VBU	vertebral body unit
AF	atrial fibrillation
MDCT	multidetector-row computed tomography
BMI	body mass index
Carina–FO	distance from the carina to the FO
AP	anteroposterior
